# Formins: Linking Cytoskeleton and Endomembranes in Plant Cells

**DOI:** 10.3390/ijms16010001

**Published:** 2014-12-23

**Authors:** Fatima Cvrčková, Denisa Oulehlová, Viktor Žárský

**Affiliations:** 1 Department of Experimental Plant Biology, Faculty of Sciences, Charles University, Viničná 5, 128 43 Prague 2, Czech Republic; E-Mail: viktor@natur.cuni.cz (V.Ž.); 2 Institute of Experimental Botany, Academy of Sciences of the Czech Republic, Rozvojová 263, 165 02 Prague 6, Czech Republic; E-Mail: denisa@email.cz (D.O.)

**Keywords:** formin, actin, microtubules, endoplasmic reticulum, Golgi, fusion proteins, AtFH4, AtFH5, AtFH16

## Abstract

The cytoskeleton plays a central part in spatial organization of the plant cytoplasm, including the endomebrane system. However, the mechanisms involved are so far only partially understood. Formins (FH2 proteins), a family of evolutionarily conserved proteins sharing the FH2 domain whose dimer can nucleate actin, mediate the co-ordination between actin and microtubule cytoskeletons in multiple eukaryotic lineages including plants. Moreover, some plant formins contain transmembrane domains and participate in anchoring cytoskeletal structures to the plasmalemma, and possibly to other membranes. Direct or indirect membrane association is well documented even for some fungal and metazoan formins lacking membrane insertion motifs, and FH2 proteins have been shown to associate with endomembranes and modulate their dynamics in both fungi and metazoans. Here we summarize the available evidence suggesting that formins participate in membrane trafficking and endomembrane, especially ER, organization also in plants. We propose that, despite some methodological pitfalls inherent to *in vivo* studies based on (over)expression of truncated and/or tagged proteins, formins are beginning to emerge as candidates for the so far somewhat elusive link between the plant cytoskeleton and the endomembrane system.

## 1. Introduction: Cytoskeleton in the Organization of Plant Endomembranes

In typical differentiated plant cells, most endomembrane organelles are literally sandwiched in a thin layer of cortical cytoplasm between the plasmalemma and tonoplast, close to each other and in an intimate contact with the cortical cytoskeleton. Common to all eukaryotes, the endomembranes are interconnected either directly or through an intensive membrane turnover (recently reviewed in [1,2]). They undergo continuous movements and dynamic remodelling, depending largely on both actin and microtubule cytoskeletons together with associated proteins including molecular motors, whose activity depends on the organization of tracks they ride on. Mechanisms of endomembrane movements may vary among eukaryotic lineages, as some of them have only been described in one or a few models (albeit absence of evidence in other lineages should not be understood as evidence of absence).

In plant cells, actin and its associated myosin motors are primarily in charge of fast relocation of cell organelles and vesicle trafficking especially to the endoplasmic reticulum (ER), with myosin XI family members (mainly XI-K) as key players in this process [3,4,5,6,7]. Organelle movement in plants is faster in areas with bundled actin cables as demonstrated on velocity of Golgi bodies entering an area with a different actin configuration [8]. However, actin may not be the only part of the cytoskeleton controlling endomembrane structure and dynamics. The metazoan Golgi apparatus is shaped by the microtubule network; it can also act *vice versa* as a microtubule organizing centre [9]. ER tubules in animal cells move mainly by “sliding” along tracks formed by a sub-population of microtubules stabilized by acetylation, and this process may be central to controlling the contacts between ER and other endomembrane compartments [10]. In plants, microtubules were shown to contribute to the formation of ER branching points, which coincide with ER to cytoskeleton anchoring sites, and also to provide anchoring sites for other endomembrane compartments or organelles [11,12]. Moreover, actin-independent extension of ER tubules along microtubules, reminiscent of metazoan ER sliding but much slower, was observed in Arabidopsis cells [13]. Both relocation and dynamics of cortical ER tubules is microtubule-dependent also in elongating giant internodal cells of characean algae [14].

Thus, involvement of both actin and cortical microtubules in the motility and shaping of ER and other endomembrane compartments may be quite a conserved feature of eukaryotic cells. However, the knowledge of actual molecules mediating the association between endomembranes and the cytoskeleton is, especially in plants, rather sparse, as the numerous proteins linking the cytoskeleton to membranes in the metazoans appear to be mostly lineage-specific. Nevertheless, a plant-specific family of NET proteins that serve to anchor actin to various endomembrane compartments including the ER has been identified recently [15,16]. A molecular mechanism responsible for actin-dependent chloroplast motility, involving an adaptor protein, CHUP1, attaching the outer plastid membrane to microfilaments [17], and two specialized kinesins required for positioning the plastids adjacent to the plasmalemma [18], has been described also. However, these proteins are likely to act only in the specific context of plastid movement, probably without any relation to the dynamics of other endomembranes.

The inventory of plant proteins mediating the connection among the endomembrane structures and the cytoskeleton is thus obviously far from complete. Additional candidates are likely to emerge especially among proteins that can associate with membranes on one hand and bind to microfilaments, microtubules, or even both cytoskeletal systems, on the other. Formins, or FH2 proteins, are an example of such a protein family. In this review we summarize the observations from both opisthokont (fungal and metazoan) and plant models, pointing to a possible role of these proteins in cytoskeleton-dependent endomembrane organization and dynamics.

## 2. FH2 Proteins as Versatile Cytoskeletal Regulators

Formins are members of an evolutionarily conserved family of multi-domain proteins defined by the presence of the conserved formin homology 2 (FH2 domain). They are ubiquitous in eukaryotes, and many species possess multiple isoforms (for a recent review see [19]). Based on FH2 domain phylogeny, plant formins can be divided into three clades, with two of them (Class I and Class II) present in angiosperms. The model plant *Arabidopsis thaliana* has 11 Class I and 10 Class II formin paralogs, with possible additional diversity generated by alternative splicing [20,21,22]. FH2 proteins are currently understood mainly as regulators of cytoskeletal dynamics, in particular since the discovery of their ability to nucleate actin [23,24]. However, their actin-related roles are not restricted to nucleation, and they also interact with microtubules in both opisthokonts and plants (reviewed e.g., in [25,26,27]). Formins may thus significantly contribute to the co-ordination of the microtubule and microfilament cytoskeletons.

Extensive domain rearrangements took place during formin evolution, resulting in incorporation of a variety of regulatory domains [22,28,29]. Nevertheless, all formins share the well-conserved hallmark FH2 domain, representing a “functional core” of the protein. The FH2 domain can form dimers capable of *de novo* nucleation of actin filaments from the barbed end by a unique mechanism referred to as processive capping [30]. However, in some formins (and some cellular contexts) it can also act as a barbed end cap (e.g., [31]), or contribute to microfilament bundling (e.g., [32]). The FH2 domain is usually preceded by an *N*-terminally located proline-rich FH1 domain, which interacts with profilin, contributes to actin assembly and stimulates FH2-mediated microfilament elongation [31]. Actin nucleation has been well documented *in vitro* also for angiosperm formins representing both Class I [33,34,35,36,37] and Class II [38,39] clades. Actin-bundling activity has been reported for several plant formins from both clades not only *in vivo*, but also *in vitro* (e.g., [33,40,41]. The enormous diversity of FH2 protein isoforms in both plants and metazoans may be, at least in part, related to their functional specialization or “fine tuning” of their activities towards actin (and possibly also towards other interaction partners). Indeed, some of the 21 *A. thaliana* formins were documented to exhibit specific expression patterns, as well as varying biochemical parameters (see [42]). The 15 human formins also vary widely in their molecular structure, biochemical activities, and tissue-specific expression [43,44].

While the actin-related functions of formins depend on the presence of the FH2 domain, the many documented interactions between formins and microtubules do not share a common molecular mechanism. Even in plants, multiple modes of FH2 protein–microtubule association must exist, since the specific GOE domain responsible for microtubule interaction in a subset of Class I formins [45] is absent in Class II formins that also bind microtubules, at least some of them with direct involvement of the FH2 domain [38,41,46,47,48].

## 3. Formins Can Associate with Cellular Membranes

Multiple mechanisms appear to account also for the observed or predicted membrane association of many FH2 proteins. At least some of them operate in every eukaryotic lineage studied so far. Binding to peripheral or integral membrane proteins is a common means of attaching formins to membranes (reviewed in [49]). The DRFs (Diaphanous-related formins), regulated by interaction with RHO clade (Rho, Rac and Cdc42) small GTPases [50] that themselves attach to membranes thanks to post-translational hydrophobic modifications, provide probably the most notorious example. Some of the DRFs, including the prototype *Drosophila* Diaphanous protein, can also directly bind membrane phosphoinositides while bound to RHO. Such a cooperative mechanism of membrane attachment contributes to protein localization to distinct membrane domains [51]. The RHO–DRF system thus provides a paradigmatic example of a membrane-associated protein selectively localizing to a distinct subset of cellular membranes, a concept important for understanding possible roles of formins in the endomembrane system.

**Figure 1:**
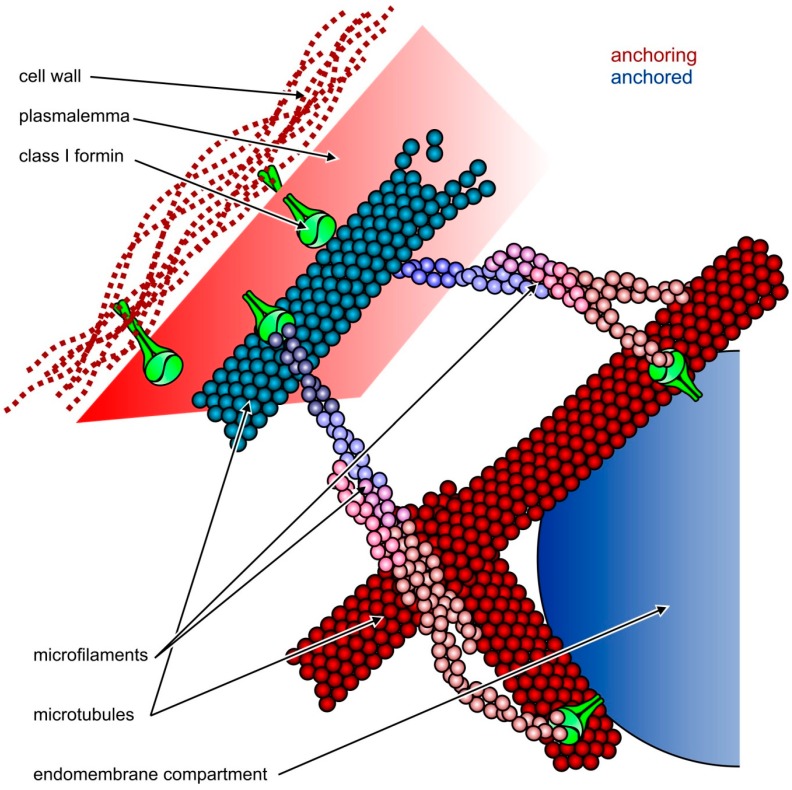
Possible roles of Class I plant formins such as AtFH8 (shown in green) in the organization of cytoskeletal and membrane structures. Relatively static structures shown in shades of red serve as a “dock” for anchoring more dynamic ones shown in blue. **Top**: formins can attach both microfilaments and microtubules to the plasmalemma, which is linked to the cell wall through integral membrane proteins, including the formins themselves (modified from [45]); **Bottom**: attachment of endomembrane compartments, such as the ER or secretory vesicles, to the cytoskeleton may be mediated by formins. While Class II formins cannot anchor the plasmalemma to the cell wall, they may also participate in endomembrane-cytoskeleton interactions as long as they bind to the membrane itself or to some integral or peripheral membrane proteins.

While DRFs *sensu stricto* are only found in animals [22], regulation by RHO, mediated by conserved GBD/FH3 motifs, is a common feature of many (though not all) FH2 protein clades, including the typical formins of fungi, and apparently is of ancestral origin [28]. At least in one case (the non-angiosperm plant Class III formins), an independent mechanism of RHO interaction, based on a domain related to conserved RhoGAPs, has been proposed [22]. However, there is no evidence so far that angiosperm formins interact directly with RHO clade GTPases (*i.e.*, the Rop–RHO of plants–family members). Nevertheless, plant formins can associate with membranes by other means (see [52]). While typical Class I angiosperm formins are transmembrane proteins with an *N*-terminal signal peptide and a single membrane-spanning helix [53,54], the most frequent domain organization of Class II formins features a PTEN-like domain whose phosphatase activity was lost by a point mutation affecting the active site, proposed to bind phosphoinositides [21]. For a *Physcomitrella patens* homologue its ability to bind PI(3,5)P_2_ was experimentally proven [55]. Thus, typical representatives of both angiosperm formin clades are capable of mediating association of both microtubules and microfilaments with membranes, and in case of Class I formins, which have an extracytoplasmic domain, also with the cell wall (Figure 1).

Most functional studies of formins in fungi and metazoa have focused on their role in cell polarity, which naturally brought attention to their functions in the cell cortex. Cortical localization of a membrane-associated protein, however, does not necessarily mean plasmalemma localization, since endomembrane structures (including, but not limited to, numerous endocytotic and exocytotic vesicles) are present at the cell cortex in most cell types. Indeed, evidence for association of FH2 proteins with intracytoplasmic structures that may involve membranes can be found upon closer inspection in many cases, as discussed below.

## 4. Fungal Formins Participate in Endomembrane Organization

Studies in yeasts and filamentous fungi suggest possible association between typical (RHO-regulated) fungal formins and endomembrane structures. In the budding yeast, *Saccharomyces cerevisiae*, the formin Bni1 is a part of the polarisome, a multiprotein cortical structure associated with sites of polarized cell growth [56]. Bni1 also localizes to polarized sub-cortical spots in the nascent bud in an actin and RHO GTPase-dependent manner [57]. Subsequent higher resolution observations showed that Bni1 associates with motile cytoplasmic speckles whose movement depends on actin cables, while the related formin Bnr1 decorates nearly static cortical structures at the bud neck [58]. While these studies do not directly address possible association between budding yeast formins and endomembranes, their results do not exclude such interpretation. Intriguingly, Bni1 together with several other polarisome subunits was recently shown to modulate the organization of the cortical ER in yeast buds through its role in the regulation of the MAP kinase Slt2 that is involved in cell wall integrity signalling [59].

More direct support for possible participation of FH2 proteins in fungal endomembrane organization comes from studies in dimorphic yeasts such as Candida, from filamentous fungi, or from budding yeasts’ mating projections (“shmoos”) that share some characteristics of tip-growing fungal hyphae [60]. In these systems, a membrane vesicle-rich, cytoskeleton-associated structure historically termed the Spitzenkörper and containing homologs of some, though not all, of the polarisome components [61,62,63] is found inside the growing tip of the tubular hypha or cell projection. Remarkably, FH2 proteins exhibit a pattern of intracellular localization and dynamics characteristic of Spitzenkörper components in several systems, including *Aspergillus nidulans*, where green fluorescent protein (GFP)-tagged formin SEPA localizes, besides future septation sites, to a dynamic “cap” on top of a cytoplasmic region rapidly staining by FM4-64 (indicating rapid membrane turnover), which is a hallmark pattern for Spitzenkörper proteins [64]. A similar localization and dynamics has been reported also for the *Candida albicans* Bni1 homolog [65] and even for budding yeast Bni1 during shmoo development [60].

Two additional lines of evidence link formins to the endomembrane system in fungi. In *Candida albicans*, the Golgi apparatus undergoes extensive remodelling upon transition between the yeast and hyphal growth phase. This event requires both actin and the Bni1 formin, as documented by pharmacological and mutant studies [66]. Last but not least, in the fission yeast *Schizosaccharomyces pombe*, Sec3, a component of the evolutionarily conserved Exocyst complex involved in targeting of secretory vesicles, was recently shown to interact physically with the formin For3. This partnership is required for proper localization of this formin (but not other polarisome components) at cell periphery and for correct formin-mediated actin cable assembly. Mutants defective in Sec3 also exhibit impaired endocytosis, albeit it is unclear if this phenotype is related to the For3 function [67]. While authors of this study presume that the relevant interaction between Sec3 and For3 takes place at the plasmalemma, earlier data show that For3 locates to intracytoplasmic speckles in the cell cortex, similar to those reported for budding yeast [68]. In any case, these findings place the For3 formin at the crossroads between actin organization and membrane turnover, the latter being inseparable from endomembrane dynamics. Remarkably, budding yeast Bem1, a scaffold protein known to interact with formins, was recently reported to bind another exocyst subunit, Exo70 [69], showing that at least in fungi the connection between formins and the exocyst is a general phenomenon.

## 5. Formins and Endomembranes: Evidence from Metazoans

In metazoan cells, endomembrane-related roles of the “classical” DRFs and related RHO-interacting formins were studied mainly in the context of the mammalian Golgi apparatus rearrangements. The congregation and dispersal of Golgi stacks depends on microtubules, but also involves actin-dependent motor activity and reorganization of Golgi-associated actin network. Formins have been implicated in both directions of Golgi remodelling, indicating that their participation is apparently paralog- and/or cell type-dependent.

In cultured mammalian lymphocytes, depletion of the RHO-regulated formin FMNL1 by RNAi results in massive accumulation of cytoplasmic actin filaments accompanied by Golgi fragmentation. Re-expression of the FMNL1γ splicing isoform that localizes to the Golgi complex, but not of other isoforms, rescues this phenotype in a manner requiring actin binding to the FH2 protein [70]. Mutational or pharmacological activation of RHO in human HeLa cells also results in similar Golgi fragmentation into “mini-stacks”. This process requires actin, microtubules and a functional homolog of the prototype DRF mDia1, while the requirement for RHO can be bypassed by expression of constitutively active mDia1. Remarkably, mDia1 was also shown to localize to Golgi-derived membrane vesicles and apparently contributes to the regulation of their formation [71]. This protein also plays a central role in the assembly of specialized membrane-anchored actin tracks for delivery of secretory vesicles to the plasma membrane in pancreatic exocrine cells, further supporting its role in the final step of exocytosis [72].

INF2 (inverted formin 2), a RHO-interacting metazoan formin that exhibits an unusual “inverted” domain structure with long *C*-terminal extensions, and containing the WASP homology 2 (WH2), domain characteristic of interactors of the Arp2/3 actin nucleation complex, was found to decorate the ER in cultured Swiss 3T3 fibroblasts due to *C*-terminal prenylation that allows its direct peripheral attachment to membranes [73]. However, in some other cell lines this protein remains cytoplasmic. The cause of this diversity was traced back to the existence of two splicing isoforms of INF2 with variant *C*-ends, expressed in varying ratios among the studied cell lines; the cytoplasmic isoform lacks the prenylation site. Interestingly, RNAi-mediated knock-down of the non-prenylated INF2 isoform resulted in Golgi dispersal (similar to the above-described consequences of FMNL1 depletion or mDia1 activation), which can be prevented by latrunculin B treatment, suggesting that cytoplasmic INF2, which is often enriched in peri-Golgi cytoplasm, also participates in actin-dependent endomembrane organization [74]. The prenylated INF2 isoform appears to mediate actin assembly on the ER surface, which is required for a key step in endoplasmic reticulum-mediated mitochondrial division (ERMD), namely the recruitment of dynamin on the surface of ER-associated mitochondria [75,76]. Interestingly, a close relative, INF1, was shown to induce, besides actin-microtubule co-alignment, formation of acetylated microtubule bundles [77], with possible implication for microtubule sliding-mediated ER movements (see above).

Investigation of mechanisms that localize formins to the metazoan endomembrane system led to the discovery of a novel, and somewhat surprising, mechanism of co-translational protein addressing. In contrast with the above-described localization of mDia1 to the Golgi apparatus of cultured mammalian cells [71], chicken Dia1 (cDia1/DIAPH1) was found to associate with perinuclear ER in embryo-derived fibroblasts. It is at present not clear whether the different localization of the two DRFs is due to different properties of the formin itself or of the studied cells. The ER localization mirrors cDia1 mRNA distribution, does not depend on either actin or microtubules, but requires successful translation of the formin’s RHO-interacting domain, which apparently binds the membrane-associated RHO GTPase as soon as it exits the ribosome [78]. The somewhat puzzling lack of actin or microtubule requirement for the perinuclear localization may be explained by the finding that translation and RHO binding to the nascent polypeptide takes place immediately after the mRNA leaves the nucleus [79]. In the plasmalemma, fine-tuned RHO GTPase-independent subcellular targeting of mouse formin mDia1 is also mediated by its direct interaction with phospholipids, since the formin induces strong clustering of PI(4,5)P_2_ prior to its insertion into the membrane [80]. Similar mechanisms might also participate in localizing at least some formins into distinct regions of endomembranes.

Thus, association with endomembrane compartments and/or functional involvement in their dynamics is a common feature of several metazoan formins. A variety of molecular mechanisms can ensure targeting of these proteins to their corresponding compartments. Post-translational modifications, alternative splicing, interaction partners, and additional (mostly not yet characterized) organism- or cell type-specific factors all contribute to the choice of role a particular formin will fulfil. While some of the mechanisms responsible for endomembrane-related formin functions are lineage-specific (such as those involving the metazoan-specific Spir actin nucleator [81]), others may be conserved across eukaryotes.

## 6. Membrane-Associated Plant Formins: No Longer Only at the Plasmalemma

Until recently, most of the research addressing membrane-related functions of plant formins focused on the roles of Class I (transmembrane) angiosperm formins in the cortical cytoskeleton-plasmalemma-cell wall continuum. Using mainly *C*-terminal fluorescent protein fusions *in vivo*, in some cases with confirmation by immunostaining, localization in the cell cortex was documented for several of these proteins, including AtFH1, the major housekeeping Class I formin in Arabidopsis, whose mobility in the plasmalemma is restricted by the cell wall [40], AtFH4 and AtFH8, both accumulating at transversal cell-to-cell boundaries in the Arabidopsis rhizodermis and root cortex [34]. AtFH5 was found at the nascent cell plate, which, however, could be viewed not only as prospective plasmalemma but also as an endomembrane compartment of a kind [35], and AtFH6 decorates the plasma membrane of giant cells in nematode-induced galls [82]. However, cortical localization may involve not only the plasmalemma but also adjacent endomembrane compartments, including small exo- and endocytotic vesicles, endosomes, TGN/Golgi or the cortical ER, which can hold a molecular subpopulation of the protein in addition to that residing at the plasmalemma. Unless interaction with the cell wall (as in the case of AtFH1 [40]), or co-localization with a plasmalemma marker is proven, or very sensitive imaging techniques are used, plasmalemma and sub-plasmalemma localization may not be reliably distinguishable *in vivo*.

**Figure 2:**
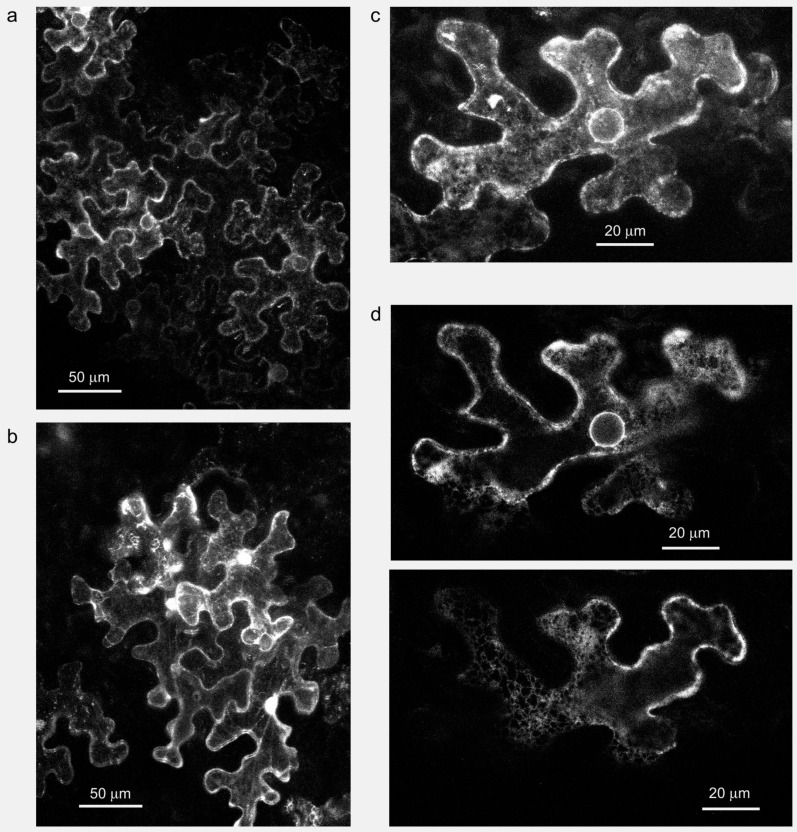
Arabidopsis Class I formin AtFH5 (At5g54650) can associate with the nuclear membrane and ER in the epidermis of *N. benthamiana* leaves after Agrobacterium-mediated transient transformation with a plasmid carrying full length AtFH5 fused to the *C* end of GFP under the control of the 35S promoter. (**a**) Maximum projection of a stack of confocal images of epidermal cells expressing GFP:AtFH5, 5 days post-transformation; (**b**) Control cells expressing free GFP showing cytoplasmic and nuclear localization, 6 days post-transformation (maximum projection); (**c**) Detail of a cell expressing GFP:AtFH5, 4 days post transformation (maximum projection); and (**d**) Two optical sections of the cell presented in (**c**), showing localization in the nuclear membrane (**top**) and in the sub-cortical ER (**bottom**).

Indeed, AtFH1, reported to accumulate in tobacco pollen tube plasmalemma upon ectopic heterologous overexpression, decorates also some intracytoplasmic structures under these conditions [83]. Remarkably, Arabidopsis mutants lacking AtFH1 exhibit impaired endocytosis, suggesting a direct or indirect participation of this formin in membrane trafficking [84]. AtFH5, which is naturally expressed in pollen, localizes both inside growing pollen tube tips and in their cortex when *C*-terminally GFP-tagged [85], and overexpression of GFP-tagged derivatives of the related pollen formin AtFH3 also resulted in a cytoplasmic signal [37]. Such observations suggest a possible association of at least a subpopulation of Class I formins with secretory or endocytotic pathway compartments. Indeed, fluorescent protein-tagged AtFH4 was upon closer observation found to localize to the cortical ER and participate in its co-alignment with microtubules [45], while AtFH8 was observed predominantly at the cell plates of dividing root cortex cells and at the nuclear envelope, with weaker signals either in the cytoplasm or the ER, although the quality of the published images does not allow distinguishing between these locations [47]. Localization to the nuclear membrane, and, to a lesser extent, the cortical ER, was also seen in *Nicotiana benthamiana* leaf epidermis transiently transformed using the p19 enhancer system [86] with constructs expressing *N*-terminally GFP-tagged AtFH5, *i.e.*, a derivative of this formin that should not enter the secretory pathway ([87]; Figure 2). In this case, the fluorescent protein may become localized through interactions with other membrane proteins, including possibly dimerization with related endogenous formins.

**Figure 3:**
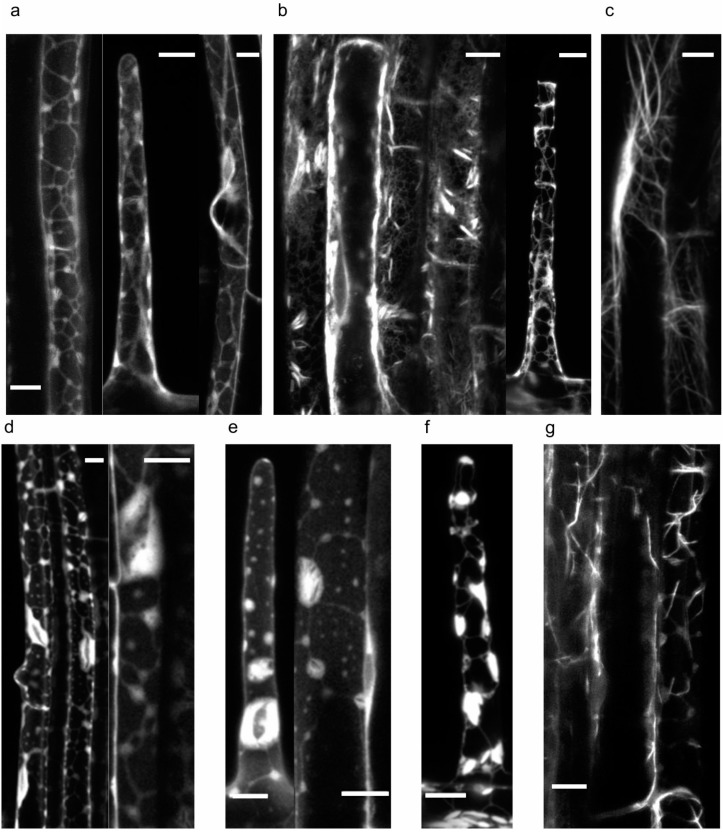
Arabidopsis Class II formin AtFH16 (At5g07770) associates with the ER in the rhizodermis of young seedlings stably transformed with a plasmid carrying full-length AtFH16 C, terminally tagged with GFP under the control of the 35S promoter. (**a**) From **left** to **right**: AtFH16:GFP in a root hair, an atrichoblast, and a bulge-stage trichoblast; (**b**) The ER marker GFP:HDEL in a control line shows a similar, though more detailed, pattern, with some bright ER bodies. **Left**—a segment of rhizodermis, **right**—a root hair; (**c**) The actin cytoskeleton, visualized by GFP:FABD in the rhizodermis of another control line, exhibits a different pattern; (**d**–**g**) Sister seedlings after LatB treatment (10 μM for the time indicated). (**d**) AtFH16:GFP after 2 h; (**e**) AtFH16:GFP after 4 h; (**f**) GFP:HDEL after 2 h; (**g**) GFP:FABD after 2 h (note actin fragmentation). Scale bars represent 10 μm. Modified from [88].

Angiosperm Class II formins lack obvious means for insertion into the membrane, and in general are less well characterized than their Class I counterparts. A moss (*P. patens*) member of this clade, associated with membranes through its PTEN-like domain, co-localizes with the FM4-64 membrane marker in the tip of growing protonemata in a pattern suggestive of possible endomembrane association [55]. However, it has to be stressed that the observed structures, although located in the apical dome of tip growing cells, bear only very superficial similarity to the above-discussed fungal Spitzenkörper, as they do not exhibit the distinctive pattern of separate domains of formin and FM4-64 localization. Another typical (*i.e.*, PTEN-like domain-containing) Class II formin, the Arabidopsis AtFH14, which can bind microtubules, localizes predominantly to the preprophase band, mitotic spindle and the phragmoplast when heterologously expressed in cultured tobacco BY2 cells, although the phragmoplast pattern does not exclude contribution of membrane structures [46]. AtFH16, a microtubule-binding Class II formin that lacks the PTEN-like domain, was found to decorate microtubules in transiently transformed onion epidermis [41] and in *Nicotiana benthamiana* leaves, where, however, additional intra-cytoplasmic structures reminiscent of the ER were also observed upon transformation with GFP-tagged AtFH16 or its deletion derivatives. However, in stably transformed Arabidopsis plants this protein decorates the ER, and pharmacological experiments have documented that its localization is sensitive to anti-actin drugs such as latrunculin B (LatB) to a similar extent as that of the ER ([88]; Figure 3).

## 7. Conclusion: Time to Look for Formin Functions in Plant Membrane Trafficking

Convincing evidence thus shows that participation in endomembrane dynamics is a common function for formins in opisthokonts. Furthermore, in plants, at least some representatives of both angiosperm-specific formin clades associate with compartments of the endomembrane system. More such examples are likely to emerge once we start looking for them. While localization to endomembrane structures *per se* does not prove a function in membrane dynamics, it is a necessary prerequisite for such a role.

Some data supporting involvement of plant formins in membrane organization and movements already exist. Overexpression of several formins, either occurring naturally, as in the case of AtFH6 upregulation in nematode-induced galls [82], or experimentally induced (e.g., ectopic expression of AtFH1 in pollen [83], overexpression of AtFH8 in rhizodermis [36], or moderate overexpression of AtFH5 in pollen [85]) correlates with obvious stimulation of cell expansion or loss of its polarity, which must involve membrane trafficking. Interestingly, massive overexpression of FH5 inhibits growth of pollen tubes [85]. Loss or impairment of formin function also leads to phenotypic effects that include altered membrane trafficking. Mutation of AtFH5 causes a cytokinesis defect [35], and expression of a dominant negative mutant of AtFH8 inhibited root hair development [34]. Functional impairment of AtFH1 by mutation or treatment with SMIFH2, a small molecule inhibitor of FH2 domain activity [89], results in altered cell expansion in both rhizodermis and cotyledon pavement cells [84,90]. Mutations of a rice Class II formin cause a complex developmental phenotype also involving altered cell growth [38,48], albeit in this case participation of phytohormone (especially auxin) signalling, a process that also to a large extent relies on membrane trafficking (for a recent review see [91]), can be expected.

In the current perspective, such phenotypic effects tend to be understood as secondary to alterations in cytoskeletal structure and function. However, at least some of them may be reflecting also a direct participation of formins in endomembrane organization and dynamics. Such an interpretation might perhaps be viewed as contrary to the time-proven Occam’s razor rule. Nevertheless, in the light of the above summarized evidence from non-plant lineages we believe that the possibility of formins also in plants directly contributing to membrane trafficking is very real, and that further experimental research should be undertaken to test this hypothesis.
